# Verbal instructions override the meaning of facial expressions

**DOI:** 10.1038/s41598-018-33269-2

**Published:** 2018-10-09

**Authors:** Florian Bublatzky, Pedro Guerra, Georg W. Alpers

**Affiliations:** 1 Department of Psychosomatic Medicine and Psychotherapy, Central Institute of Mental Health Mannheim, Medical Faculty Mannheim/Heidelberg University, Mannheim, Germany; 2 Clinical Psychology and Biological Psychology and Psychotherapy, Department of Psychology, School of Social Sciences, University of Mannheim, Mannheim, Germany; 3 University of Granada, Department of Personality, Granada, Spain

## Abstract

Psychological research has long acknowledged that facial expressions can implicitly trigger affective psychophysiological responses. However, whether verbal information can alter the meaning of facial emotions and corresponding response patterns has not been tested. This study examined emotional facial expressions as cues for instructed threat-of-shock or safety, with a focus on defensive responding. In addition, reversal instructions were introduced to test the impact of explicit safety instructions on fear extinction. Forty participants were instructed that they would receive unpleasant electric shocks, for instance, when viewing happy but not angry faces. In a second block, instructions were reversed (e.g., now angry faces cued shock). Happy, neutral, and angry faces were repeatedly presented, and auditory startle probes were delivered in half of the trials. The defensive startle reflex was potentiated for threat compared to safety cues. Importantly, this effect occurred regardless of whether threat was cued by happy or angry expressions. Although the typical pattern of response habituation was observed, defense activation to newly instructed threat cues remained significantly enhanced in the second part of the experiment, and it was more pronounced in more socially anxious participants. Thus, anxious individuals did not exhibit more pronounced defense activation compared to less anxious participants, but their defense activation was more persistent.

## Introduction

The ability to communicate about future events and their potential consequences is highly advantageous for gaining benefits and avoiding danger. Such vital information can be transmitted using non-verbal communication (e.g., facial expressions, body posture)^1,2^, but also via verbal or written instructions (e.g., ‘beware of …’). Both sources of information – visual facial expressions and language – have been shown to effectively modulate the activity of motivational systems in the brain, to prepare for adequate responding in a given situation^3–7^. However, to what degree facial expressions and verbal instructions interact in guiding person perception and social behavior is not well-understood.

There is a strong body of research examining the role of facial expressions and their capability to mediate perceptual processing and behavioral responding in social situations. Viewing threat-related emotional expressions – such as fear or anger – has been shown to be associated with enhanced activation of the autonomous nervous system and speeded behavioral responding^8–10^. Similarly, happy facial expressions have been suggested to receive preferential access to attentional processing resources compared to neutral faces. For instance, happy faces have been linked to better detection rates^11,12^ and facilitated electrocortical processing (e.g., LPP component)^13^. However, observing unknown people who smile might also be more ambivalent as their actual intention remains uncertain^5,14^. Together, these psychophysiological response patterns have been suggested to reflect the workings of basic motivational systems that organize behavioral approach or withdrawal (e.g., defense behavior)^15,16^. Accordingly, facial expressions of emotion are presumed to be evolutionarily prepared to receive more attentional resources and prime emotion-specific motor-behavioral responding^10,17^.

Language is another evolutionary prepared communication system. Affective language, such as insults or compliments, is especially effective at catching the listeners’ attention. This is particularly evident when information directly refers to the listener or reader^18–20^. Accordingly, verbal instructions about imminent aversive events (threat-of-shock) effectively enhance perceptual processing^21–23^, defensive activation^4,7,24,25^, and modulate overt behavioral responding (e.g., in decision-making tasks)^26,27^. Importantly, this verbal information does not need to be substantiated by first-hand experiences of the anticipated aversive events. For instance, despite the lack of aversive reinforcement, instructed threat contingencies are very resistant to extinction even across several days^28^. However, verbal instructions can reverse threat expectancies, for instance, when an instructed threat cue is newly learned as a safety cue^29–31^. Given the centrality of threat perception for interpersonal relations and social behavior, associations between threatening events and facial information might be malleable and flexibly change according to social settings.

The present study examined the joint impact of visual and verbal affective information on defensive responding. To this end, pictures of happy and angry facial expressions were verbally instructed as cues for the threat of electric shocks or safety. As dependent variables, we chose both somatic (startle reflex) and autonomic indices (skin conductance response [SCR] and heart rate [HR]), which have been shown to be sensitive to facial expressions and verbal instructions^5,9,24^. In addition to physiological measures, we also obtained subjective ratings about the perceived threat, affective valence, and emotional arousal. Following two previous studies that used pictures of affective scenes as shock cues (i.e., pleasant and unpleasant IAPS pictures)^24,32^, threat instructions were predicted to change the inherent valence of facial expressions. This should be evinced by threat-potentiated startle reflex, enhanced SCRs, and potentiated initial HR deceleration, as well as higher threat ratings for threat-of-shock relative to safety cues^4,7,24,25^.

Focusing on the interaction of facial emotions and verbal threat/safety instructions, the congruency of affective information (e.g., an angry face instructed to signal shock threat compared to safety) was of particular interest. According to the motivational priming theory^15,16^, an interaction of threat/safety instruction by facial expression was expected: When serving as a threat cue, inherently unpleasant stimuli (i.e., angry faces) will elicit more pronounced defensive responding than inherently pleasant stimuli (i.e., happy faces). Alternatively, incongruent information might be particularly effective in guiding defense activation to unknown people. For instance, a smiling person instructed to signal shocks may be considered as particularly dangerous^14^, with implications for social interactions and behavior towards this person (e.g., impression formation, social bonding)^33,34^.

Also, we expected to gain insight into the malleability of instruction effects by examining reversal learning. Reversal learning reflects the shift of threat associations from one stimulus to another, with the concurrent inhibition (previous threat cue becomes safe) and acquisition of threat contingencies (a previous safety cue becomes threatening)^35^. To this end, a second experimental block was preceded by additional instructions, which aimed at reversing previously learned threat/safety contingencies (e.g., from threat to safety or vice versa)^36,37^. Here, it is of interest whether the impact of reversal instructions on fear extinction learning depends on prepared learning mechanisms in person perception^36^. Specifically, we examined whether threat effects were more stable when angry (relative to happy) faces served as reversed safety cues (i.e., previously cueing threat). Moreover, we predicted that pleasant facial expressions might be less effective as a threat cue^37^, or that they are more quickly associated with safety in the reversal test.

## Methods

### Participants

Sample size was determined using G*Power^38^, which indicated that N = 40 was required to detect all relevant physiological effects at a medium effect size (f = 0.25, α error = 0.05, power = 0.8, and assumed correlation of repeated measures = 0.4). This stop rule for data collection was also in line with previous startle studies using emotional facial expression and threat-of-shock instructions^3,24,30^. Forty healthy volunteers (10 males) were recruited from the students of the psychology department at the University of Mannheim. Participants’ age ranged between 17 and 52 (*M* = 22.7, *SD* = 7.1) and the sample was within the normal range of state and trait anxiety (STAI, *M* = 35.3 and 35.1, *SD* = 5.8 and 8.8), social anxiety (SPIN, *M* = 10.9, *SD* = 8.2), and depression (BDI, *M* = 5.9, *SD* = 6.5). All participants were informed about the general study procedure before informed consent was obtained. The ethical review committee of the University of Mannheim approved all utilized procedures and methods. Participants received course credits for their participation.

### Stimulus materials and presentation

Face pictures were selected from the Karolinska Directed Emotional Faces (KDEF^39^), a well-established stimulus set providing pictures of human facial expressions of emotion. Sixteen actors (eight females) displaying happy, neutral, and angry facial expressions, were selected based on visual inspection (i.e., seven raters agreed upon the clarity and recognizability of facial expressions). The KDEF face identifiers were af01, af07, af09, af11, af19, af20, af22, af29, am02, am03, am07, am08, am10, am13, am14, and am25.

**Figure 1:**
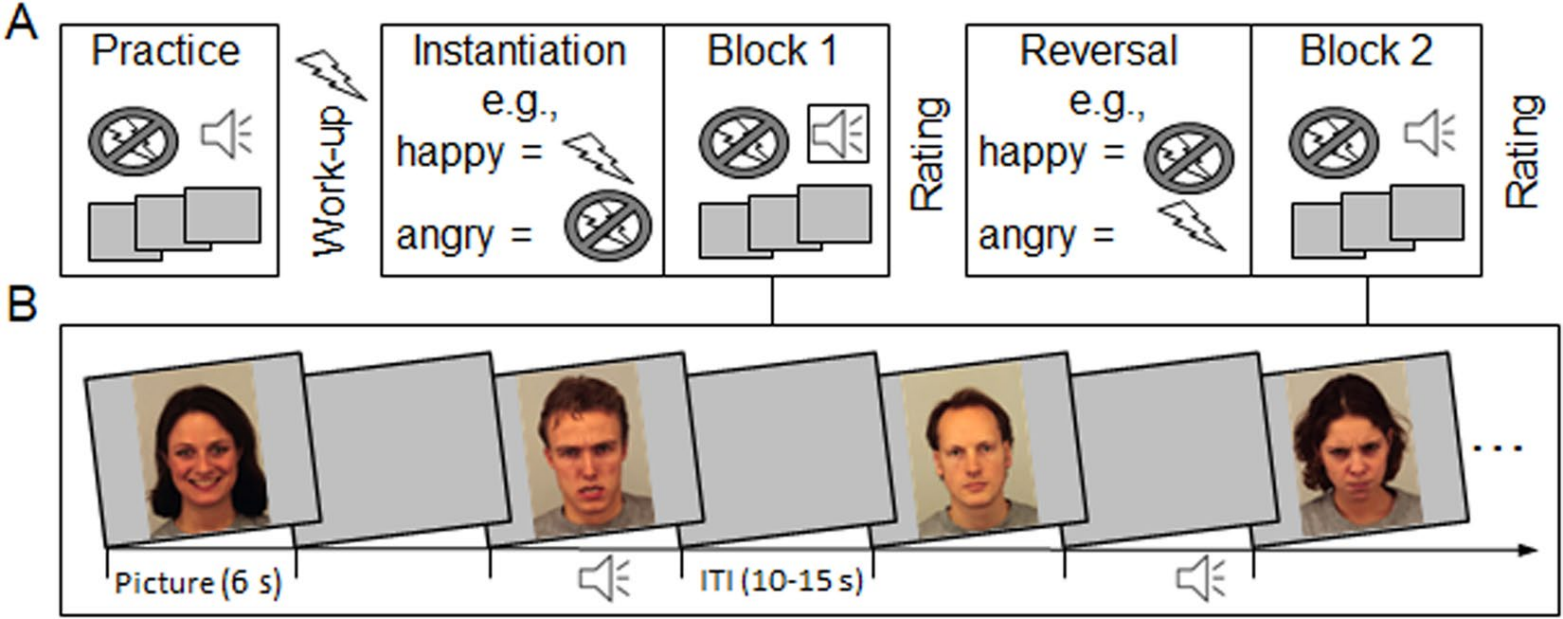
Schematic illustration of the experimental procedures and stimulus presentation. (**A**) After a brief practice run and shock work-up procedure, participants were verbally instructed that one particular emotional facial expression serves as a cue for threat-of-shock (e.g., happy) or safety (e.g., angry) and the first experimental block started (instantiation). Preceding the second experimental block (reversal), a verbal reversal instruction stated that now threat and safety contingencies are reversed (e.g., now angry faces cue threat and happy cue safety). The order in which facial expressions cued threat or safety was tested in two groups (each N = 20 completed the happy-angry or angry-happy threat order). Please note, neutral faces always cued safety. Following each block, threat and safety cues were rated regarding valence, arousal, and perceived threat. (**B**) Within each block, face pictures displaying happy, neutral, and angry facial expressions were presented (each 6 s) with a variable intertrial interval (ITI, 10 to 15 s). Auditory startle probes were presented occasionally during pictures and ITIs, no shocks were presented during the experiment. Example pictures are taken from the KDEF (identifiers: af01has, am08ans, am10nes, and af20ans).

All 48 pictures (1024 × 768 pixels) were presented for 6 s separated by variable inter-trial intervals (ITI) ranging from 10 to 15 s to allow response recovery (see Fig. 1). To provoke the defensive startle reflex, auditory startle probes (white noise, 105 dB, 50 ms) were presented during half of the picture trials. The 48 pictures (including the 24 picture-startle trials) were evenly distributed across two experimental blocks (instantiation, reversal) and three facial expressions (happy, neutral, angry), resulting in four picture-startle trials for each experimental condition per participant. To prevent the predictability of auditory stimulation, startle probes were presented at either 4, 4.5, 5 or 5.5 s after picture onset (i.e., while the picture was still visible), and six additional startle probes (three per block) were presented during the ITI. Startle probes were presented binaurally using headphones (AKG K44 Perception) and the average lag between probes was 28.8 s.

Presentation software (Neurobehavioral Systems, Inc., Albany, CA, USA) served to control the stimulus presentation, which was pseudorandom regarding picture sequence (no immediate repetition of the same face actor, no more than three pictures of the same facial expression in a row) and regarding startle presentation (no more than two picture-startle trials in a row). Electric stimuli for the shock work-up procedure were presented using a Digitimer Stimulator DS-5 (up to 10 shocks, with maximal 10 mA, 100 ms).

### Experimental task and instructions

Participants’ task was to look at all pictures, which were presented during the two experimental blocks (instantiation and reversal; see Fig. 1). Immediately before the first block started (instantiation), participants were verbally instructed that they might receive up to three electric shocks under specific conditions. One group of participants (N = 20) was told that electric shocks might be administered whenever an angry face is presented (angry = threat) but not when they see a happy face (happy = safety). The other group (N = 20) received the opposite instruction, stating that happy facial expressions cued threat-of-shock (happy = threat), and safety condition being signaled by angry faces (angry = safety). For the second experimental block (reversal), all participants were verbally instructed that now threat and safety contingencies were reversed. Specifically, the previous threat cue becomes safe, and the previous safety cue becomes threatening. Thus, across both experimental groups, happy and angry facial expressions served equally often as instructed and reversed threat and safety cue; neutral faces always signaled safety.

### Procedure

Participants completed questionnaires on general and social anxiety and depression (State-Trait Anxiety Inventory [STAI-state/trait], Social Phobia Inventory [SPIN], Social Interaction Anxiety Scale [SIAS], Beck Depression Inventory [BDI]). Sensors for physiological recordings were attached, and an electric stimulation electrode was placed at the right upper arm. Next, a brief shock work-up procedure (without picture presentation) was carried out to ensure the credibility of the threat instruction^22,40^. To set the shock intensity individually at a level rated as “maximally unpleasant but not yet painful”, participants received up to 10 shocks with increasing intensity. Participants were then instructed that the intensity of the electric shocks given during the experiment would be equal to the most unpleasant test stimulus.

Practice trials served to familiarize participants with the picture and startle presentation procedure and to allow for initial habituation of the startle reflex. Afterward, verbal instructions regarding threat and safety contingencies were given (i.e., which facial expression signals threat-of-shock and which signals safety) and the first experimental block started (instantiation). Following this block, participants rated the hedonic valence and arousal using the Self-Assessment Manikin (SAM)^41^, and perceived threat of the facial expressions using a visual analog scale ranging from *not at all* to *highly threatening* (1 to 10). Then all participants received the instruction that threat/safety contingencies were now reversed (e.g., the threat cue becomes safe, and safety cue becomes threatening), and the second block started (reversal). Facial expressions were rated again after the reversal block. Finally, participants were debriefed. No shocks were presented during the experiment. Thus, results reflect physiological responding during the anticipation (but not experience) of electric shocks.

### Data recording and reduction

Psychophysiological measures were recorded continuously with a vAmp amplifier (BrainProducts, Munich, Germany). Startle amplitudes were derived from the electromyogram of the orbicularis muscle using two miniature Ag/AgCl electrodes. The raw signal was recorded at a 1000 Hz sampling rate and frequencies below 28 Hz and above 500 Hz were filtered out with a band-pass filter (24 dB/octave roll-off). Raw electromyogram (EMG) data were rectified and smoothed with a moving average procedure (50 ms) in VisionAnalyzer 2.0 (BrainProducts). Startle responses were scored with an automated procedure and defined as the maximum peak in the 21–150 ms time window following each startle probe. Peak amplitudes were calculated relative to a mean baseline period (50 ms preceding startle response time window)^28,42^.

As an index of phasic autonomic activation, skin conductance responses (SCRs) were recorded with Ag/AgCl electrodes (constant voltage of 0.5 V; 20 Hz sampling rate) placed at the hypothenar eminence of the non-dominant hand. SCRs to picture onset were calculated as the maximum increase in skin conductance in the interval of 1 to 6 s (relative to a 1 s pre-stimulus period). A minimum threshold of 0.02 µS was used for zero-response detection, and range and distribution corrections were applied. Phasic heart rate changes to picture onset was derived from the electrocardiogram recorded at lead II. The electrocardiogram signal was recorded at 1000 Hz, and frequencies below 0.1 and above 13 Hz were filtered. The weighted HR averages every half second were expressed in terms of differential scores with respect to a 2 s baseline period^24^.

### Data analysis and statistical design

Self-report (valence, arousal, and threat ratings) and physiological data (startle-EMG and SCR) were submitted to (2 × 2) × 2 repeated measures ANOVA, including the within-subject factors Instruction (threat vs. safety) and Block (instantiation Block 1 vs. reversal Block 2), as well as the between-group factor Order (happy-angry vs. angry-happy). The Order referred to the sequence in which emotional facial expression cued threat or safety in which experimental block. Specifically, for the happy-angry order, happy faces served as threat cue during instantiation block (Block 1), and angry faces cued threat during the following reversal block (Block 2). This was reversed for the angry-happy order, in which angry faces during Block 1 and happy faces in Block 2 cued threat-of-shock. Regarding phasic changes in heart rate, an additional factor, Time (12), was implemented to compare half-second changes after picture onset.

To examine the impact of emotional facial expression on the instantiation and reversal of threat instructions, planned comparisons focused separately on each Order (happy-angry vs. angry-happy). Please note that for reasons of brevity and to reduce the complexity of the overall design, neutral faces cued safety in both blocks and were thus excluded from the analyses of instructed and reversed threat. However, supplementary analyses were conducted to compare Facial expression (happy vs. neutral vs. angry) when serving as a safety cue (see Supplemental Material). Covariation analyses were conducted to test the impact of inter-individual differences in reported social- and trait-anxiety on defense activation.

Greenhouse-Geisser corrections were used where relevant, and the partial ƞ^2^ is reported as a measure of effect size. To control for Type 1 error, Bonferroni correction was applied for post-hoc *t*-tests.

## Results

### Self-report data

#### Threat ratings

Overall, instructed threat cues were rated as more threatening relative to safety cues, *F*(1,39) = 17.37, *p* < 0.001, η_p_^2^ = 0.31, and perceived threat decreased from the instantiation block to the reversal block, *F*(1,39) = 5.98, *p* < 0.05, η_p_^2^ = 0.13. The interaction Instruction × Block did not reach significance, *F*(1,39) = 1.47, *p* = 0.23, η_p_^2^ = 0.04, however, a significant three-way interaction Instruction × Block × Order emerged, *F*(1,38) = 56.72, *p* < 0.001, η_p_^2^ = 0.60.

**Figure 2:**
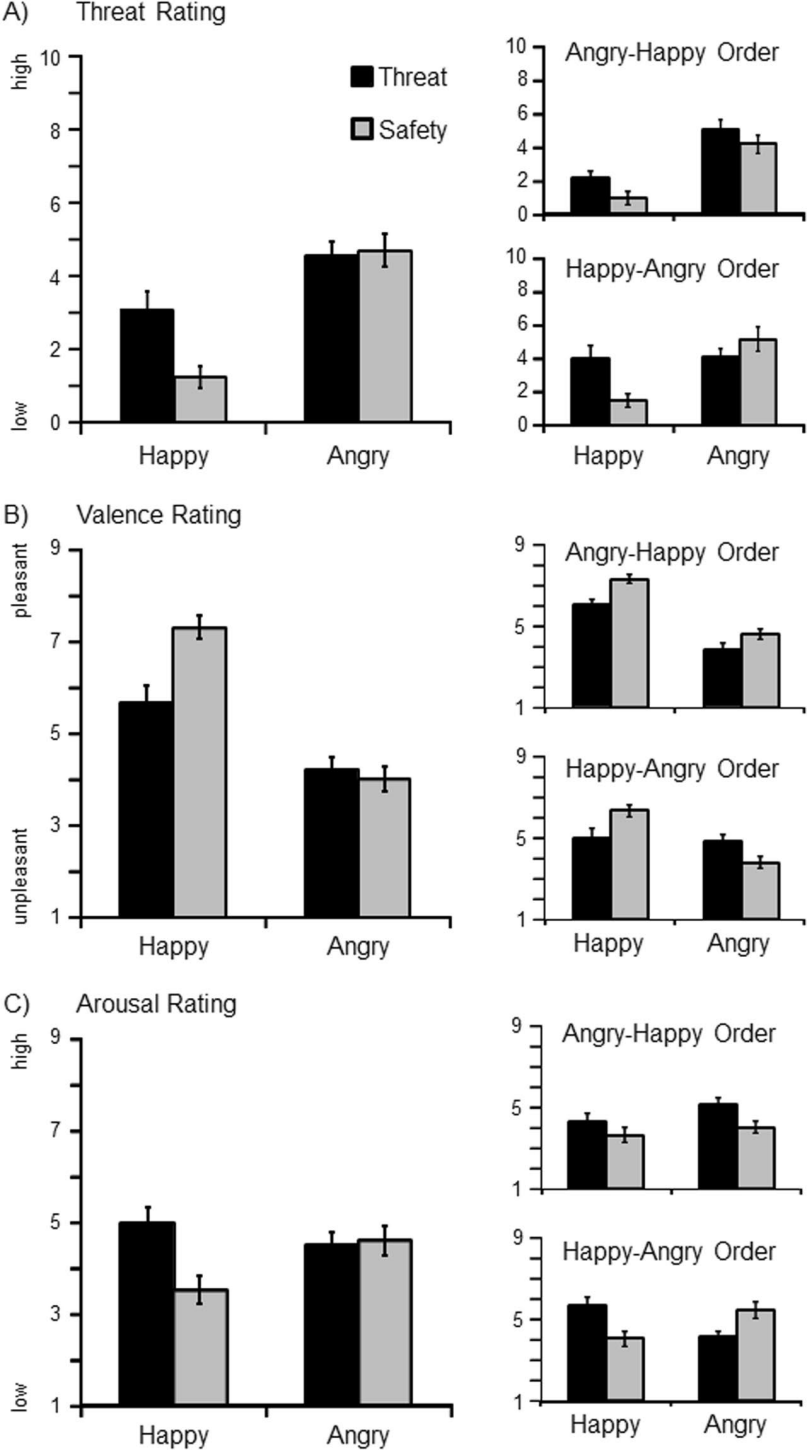
Self-reported threat (**A**), valence (**B**), and arousal (**C**) ratings as a function of facial expression (happy, angry) and instructions (threat, safety). The left side illustrates overall means (with SEM) averaged across experimental blocks and orders. On the right side, separate means are plotted for each order. The angry-happy order started with angry facial expression cueing threat during the instantiation block and happy faces cueing threat during the following reversal block. For the happy-angry order, instructed threat/safety contingencies were vice versa.

Follow-up analyses run separately for the Happy-Angry order (see Fig. 2A; Table 1) showed that instructed threat effects varied across blocks, *F*(1,19) = 21.26, *p* < 0.001, η_p_^2^ = 0.53. Specifically, pronounced threat ratings were observed for happy facial expressions cueing threat-of-shock during instantiation, *F*(1,19) = 5.23, *p* < 0.05, η_p_^2^ = 0.22, and for angry faces cueing threat in the subsequent reversal block, *F*(1,19) = 31.30, *p* < 0.001, η_p_^2^ = 0.62. Similarly, for the Angry-Happy order, an interaction Instruction × Block emerged *F*(1,19) = 35.46, *p* < 0.001, η_p_^2^ = 0.65. Separate comparisons of threat and safety cues indicate more pronounced threat ratings for angry faces during the instantiation block, *F*(1,19) = 35.04, *p* < 0.001, η_p_^2^ = 0.65, and happy expressions in the reversal block, *F*(1,19) = 16.50, *p* < 0.01, η_p_^2^ = 0.47.

**Table 1:** Mean amplitudes and standard deviations (*M*, *SD*) as a function of Block (instantiation vs. reversal), Instruction (threat vs. safety), and Order of threat instruction (Angry-Happy vs. Happy-Angry). Means are provided for the startle reflex, skin conductance responses (SCR), heart rate (HR), and ratings of the self-reported valence, arousal, and perceived threat.

Block	Instruction	Order	Startle		SCR		HR		Valence		Arousal		Threat	
			*M*	*SD*	*M*	*SD*	*M*	*SD*	*M*	*SD*	*M*	*SD*	*M*	*SD*
Instantiation (Block 1)	Threat	Angry-Happy	57.85	5.14	0.115	0.11	−2.75	1.66	3.55	1.73	5.15	1.66	5.05	2.61
		Happy-Angry	57.48	5.31	0.097	0.17	−2.75	2.71	5.05	2.48	5.80	2.14	4.05	3.61
	Safe	Angry-Happy	49.54	3.31	0.030	0.04	−0.78	1.85	7.90	1.25	3.30	2.13	0.95	1.76
		Happy-Angry	49.77	3.60	0.032	0.07	−1.30	4.36	3.50	1.67	5.45	2.19	5.20	3.08
Reversal (Block 2)	Threat	Angry-Happy	50.26	5.99	0.048	0.10	−1.64	3.28	6.30	1.75	4.15	2.08	2.15	1.95
		Happy-Angry	51.21	5.44	0.079	0.24	−2.58	2.65	4.85	1.69	3.90	1.71	4.10	2.38
	Safe	Angry-Happy	46.58	4.22	0.014	0.02	−1.33	2.62	4.50	1.54	3.75	1.62	4.23	2.46
		Happy-Angry	45.41	4.32	0.034	0.05	−1.29	2.31	6.70	1.75	3.75	1.89	1.55	1.79

Heart rate changes refer to averages across the significant time intervals from 3 to 5 s after picture onset.

#### Valence ratings

Overall, threat cues were rated as more unpleasant than safety cues, *F*(1,39) = 11.01, *p* < 0.01, η_p_^2^ = 0.22, and unpleasantness decreased across blocks, *F*(1,39) = 4.62, *p* < 0.05, η_p_^2^ = 0.11. Whereas no interaction of Instruction × Block was observed, *F*(1,39) = 1.99, *p* = 0.17, η_p_^2^ = 0.05, a significant three-way interaction was found, Instruction × Block × Order *F*(1,38) = 60. 87, *p* < 0.001, η_p_^2^ = 0.62.

Separate analysis for the Happy-Angry order (Fig. 2B) showed a significant interaction Instruction × Block, *F*(1,19) = 13.53, *p* < 0.01, η_p_^2^ = 0.42. Interestingly, happy expressions were rated as more pleasant than angry faces even when happy faces served as instructed threat cue in the instantiation block, *F*(1,19) = 11.01, *p* < 0.01, η_p_^2^ = 0.37. In the reversal block, angry faces cueing threat were rated as more unpleasant than happy faces cueing safety, *F*(1,19) = 11.56, *p* < 0.01, η_p_^2^ = 0.38. For the Angry-Happy order, a significant interaction Instruction × Block was evident, *F*(1,19) = 58.77, *p* < 0.001, η_p_^2^ = 0.76. Angry faces cueing threat were more unpleasant during the instantiation block, *F*(1,19) = 72.96, *p* < 0.001, η_p_^2^ = 0.79, and this threat effect was less pronounced when happy facial expressions served as new threat cues in the reversal block, *F*(1,19) = 11.28, *p* < 0.01, η_p_^2^ = 0.37.

#### Arousal ratings

Overall, instructed threat cues were rated as more arousing than safety cues, *F*(1,39) = 10.41, *p* < 0.01, η_p_^2^ = 0.21, and arousal decreased from the instantiation to the reversal block, *F*(1,39) = 21.90, *p* < 0.001, η_p_^2^ = 0.36. Moreover, the interaction of Instruction × Block was significant, *F*(1,39) = 4.83, *p* < 0.05, η_p_^2^ = 0.11, showing pronounced threat effects in the instantiation block, *F*(1,39) = 13.52, *p* < 0.01, η_p_^2^ = 0.26, but not in the reversal block, *F*(1,39) = 1.05, *p* = 0.31, η_p_^2^ = 0.03. This pattern did not significantly differ between experimental orders (Angry-Happy or Happy-Angry; Fig. 2C), Instruction × Block × Order *F*(1,38) = 2.91, *p* = 0.10, η_p_^2^ = 0.07.

### Startle reflex

**Figure 3:**
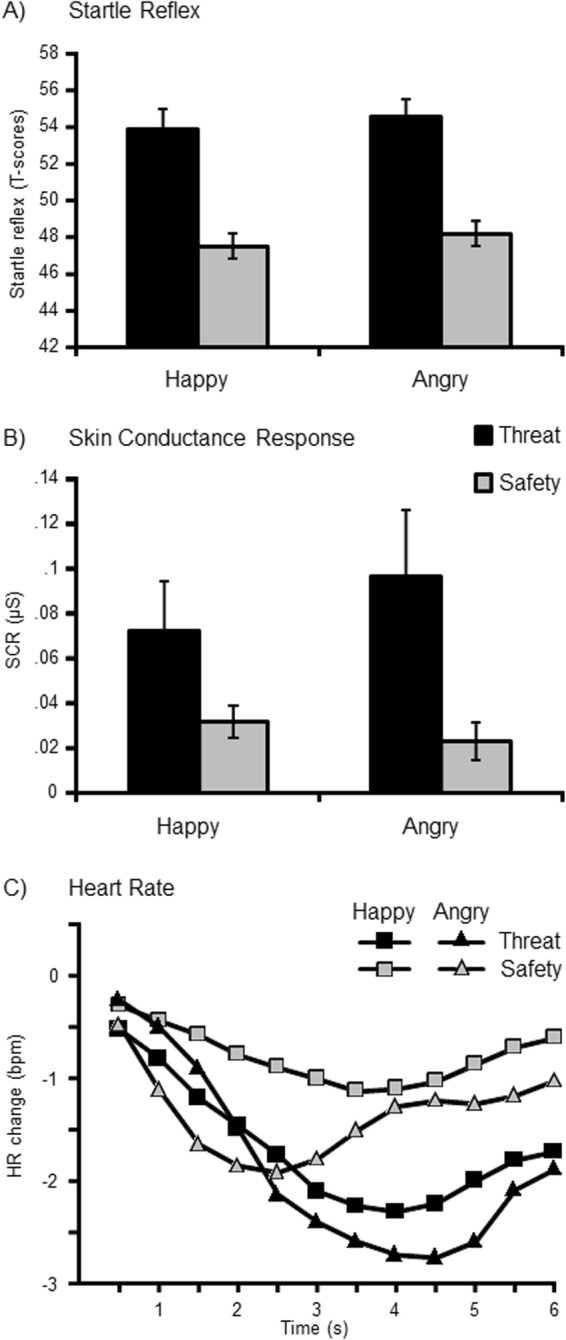
Mean responses of the startle reflex (**A**) and skin conductance (**B**) for happy and angry facial expressions serving as threat or safety cue (with SEM). Heart rate changes (**C**) are averaged every half a second after stimulus onset. As no interaction effects occurred with the sequence of instructions, averages across experimental orders (happy-angry and angry-happy) are illustrated.

**Figure 4:**
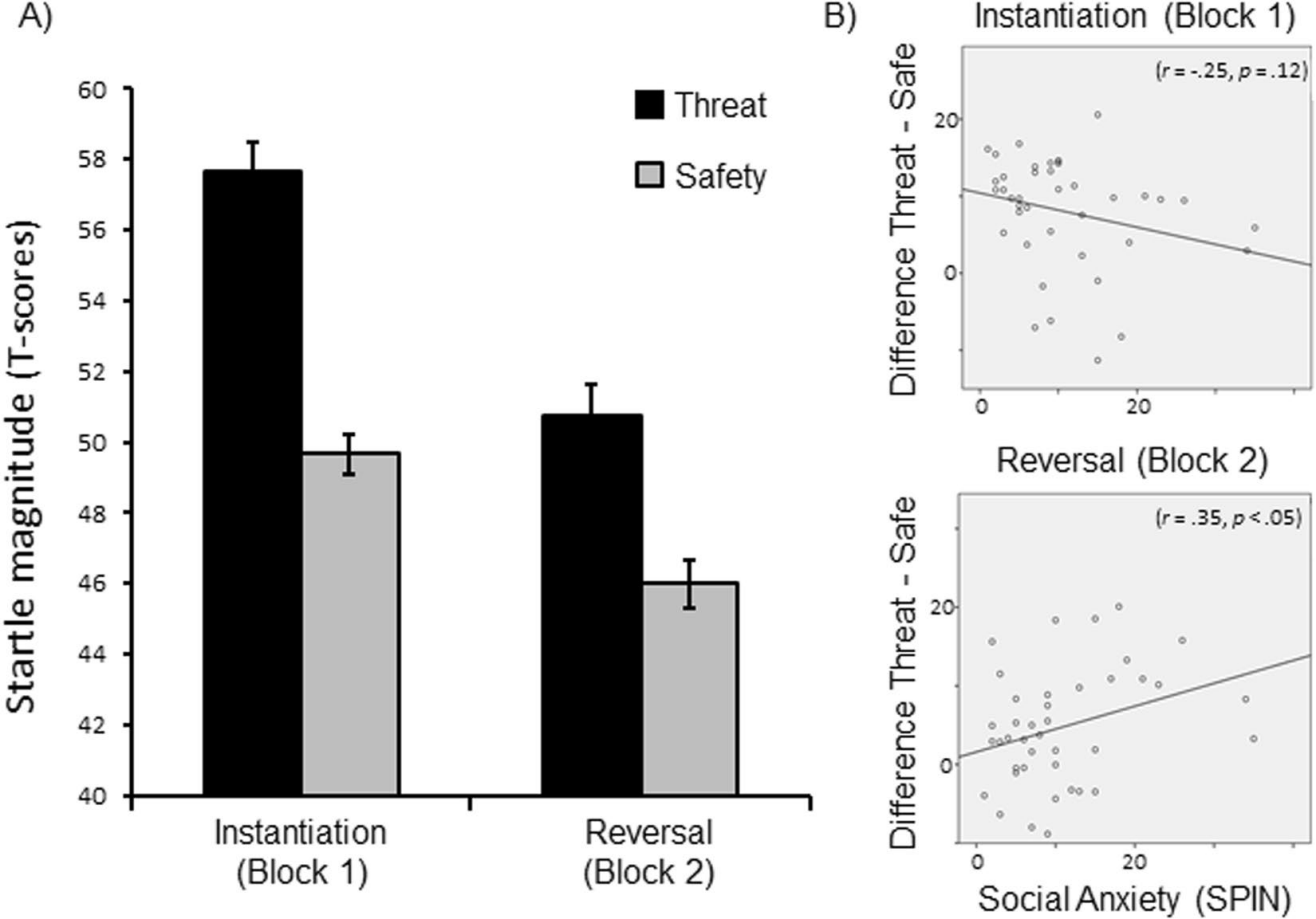
(**A**) Mean startle responses as a function of threat/safety instructions averaged across experimental blocks and orders (with SEM). Threat and safety contingencies were instantiated in Block 1 (e.g., angry faces cue threat) and reversed in Block 2 (e.g., happy faces cue threat; or vice versa). (**B**) Scatterplots illustrate the covariation between individuals’ social anxiety and threat-potentiated startle effects (differences between threat and safety) separately for the instantiation and reversal block. Overall, threat effects are malleable and stable; anxious participants reveal a more persistent pattern of defense activation after reversal instructions.

The defensive startle reflex was more pronounced when viewing threat as compared to safety cues (Fig. 3A), *F*(1,39) = 56.87, *p* < 0.001, η_p_^2^ = 0.59, and a pronounced pattern of response habituation was observed across experimental blocks (Fig. 4A), *F*(1,39) = 58.19, *p* < 0.001, η_p_^2^ = 0.60. Moreover, reflex amplitudes varied as a function of Instruction × Block, *F*(1,39) = 4.26, *p* < 0.05, η_p_^2^ = 0.10, indicating that threat-potentiation decreased from the instantiation to the reversal block. Post-hoc tests revealed pronounced differences between threat and safety cues within the instantiation block, and less markedly but still highly significant in the following reversal block, *Fs*(1,39) = 48.78 and 16.52, *ps* < 0.001, η_p_^2^ = 0.56 and 0.30. Importantly, the inherent valence of emotional facial expressions did not modulate the instantiation and reversal of threat as observed for the startle reflex, Order × Instruction × Block, *F*(1,38) = 0.73, *p* = 0.40, η_p_^2^ = 0.02. Planned follow-up tests focused on each experimental order separately.

When angry faces signaled threat during the instantiation block and served as safety cue in the subsequent reversal block (Angry-Happy order), main effects of Instruction and Block were significant, *Fs*(1,19) = 23.67 and 49.31, *ps* < 0.001, η_p_^2^ = 0.56 and 0.72. Moreover, startle amplitudes tended to vary as a function of Instruction × Block, *F*(1,19) = 3.38, *p* = 0.08, η_p_^2^ = 0.15. Separate analyses for each block revealed that angry faces as threat cue triggered highly significant threat effects during instantiation, *F*(1,19) = 24.66, *p* < 0.001, η_p_^2^ = 0.57, but only marginal threat effects were observed when angry faces served as safety cue in the reversal block, *F*(1,19) = 4.02, *p* = 0.06, η_p_^2^ = 0.17. In contrast, for the Happy-Angry order, when happy faces served as threat cue during instantiation and as safety cue in the reversal block, main effects of Instruction and Block were found, *Fs*(1,19) = 19.97 and 32.59, *ps* < 0.001, η_p_^2^ = 0.51 and 0.63. However, threat effects were not reduced across blocks when angry faces cued shock threat in the reversal block, Instruction × Block *F*(1,19) = 0.96, *p* = 0.34, η_p_^2^ = 0.05. Follow-up tests revealed pronounced threat-potentiated startle for happy faces cueing threat in the instantiation block, *F*(1,19) = 22.95, *p* < 0.001, η_p_^2^ = 0.55, which was similarly pronounced in the subsequent reversal block when angry faces cued threat, *F*(1,19) = 16.1, *p* = 0.001, η_p_^2^ = 0.46. Thus, during the reversal block, instruction effects were more resistant to extinction when angry rather than happy faces cued threat.

Exploratory analyses revealed that the overall interaction Instruction × Block varied as a function of inter-individual differences in reported social- and trait-anxiety (Fig. 4B). Specifically, significant covariation effects were observed with SPIN scores, *F*(1,38) = 8.15, *p* < 0.01, η_p_^2^ = 0.18, STAI-trait, *F*(1,38) = 7.81, *p* < 0.01, η_p_^2^ = 0.17, and marginally with SIAS, *F*(1,38) = 3.71, *p* = 0.06, η_p_^2^ = 0.09. To follow up on these interactions, correlational analyses were conducted between anxiety scores and startle amplitudes (i.e., the difference scores between threat minus safety) separately for each block. For the instantiation block, threat effects did not vary with anxiety level (*r*_trait-anxiety_ = −0.20, *p* = 0.21; *r*_SPIN_ = −0.25, *p* = 0.12; *r*_SIAS_ = −0.08, *p* = 0.61). In the subsequent reversal block, however, threat-potentiated startle was more pronounced in participants who scored higher on anxiety (*r*_trait-anxiety_ = 0.36, *p* < 0.05; *r*_SPIN_ = 0.32, *p* < 0.05; *r*_SIAS_ = 0.32, *p* < 0.05). Thus, anxious participants did not exhibit more pronounced, but more persistent defense activation compared to less socially anxious participants.

### Skin conductance responses

Enhanced skin conductance responses (SCR) were observed for threat relative to safety cues, *F*(1,39) = 9.29, *p* < 0.01, η_p_^2^ = 0.19 (see Fig. 3B). SCRs diminished over time, they were more pronounced in the instantiation block than in the subsequent reversal block, *F*(1,39) = 7.21, *p* < 0.05, η_p_^2^ = 0.16. The interaction Instruction × Block didn’t reach significance, *F*(1,39) = 2.95, *p* = 0.09, η_p_^2^ = 0.07. Exploratory follow-up analyses revealed significant threat effects during instantiation, *F*(1,39) = 20.93, *p* < 0.001, η_p_^2^ = 0.35, but not in the reversal block, *F*(1,39) = 2.31, *p* = 0.14, η_p_^2^ = 0.06. Importantly, the inherent facial valence did not modulate SCRs for instantiation and reversal of threat, Order × Instruction × Block, *F*(1,38) = 0.53, *p* = 0.47, η_p_^2^ = 0.01. Planned comparisons focused separately on each experimental order.

When angry faces served initially as threat cues, and later as safety cues (Angry-Happy order), there were significant main effects of Instruction, *F*(1,19) = 13.34, *p* < 0.01, η_p_^2^ = 0.41, and Block, *F*(1,19) = 8.43, *p* < 0.01, η_p_^2^ = 0.31, as well as a trend to an interaction Instruction × Block, *F*(1,19) = 3.55, *p* = 0.08, η_p_^2^ = 0.16. Follow-up analyses revealed threat-enhanced SCRs when angry faces cued threat during the instantiation block, *F*(1,19) = 15.15, *p* = 0.001, η_p_^2^ = 0.44, but not when happy faces cued threat in the reversal block, *F*(1,19) = 2.63, *p* = 0.12, η_p_^2^ = 0.12. For the Happy-Angry order, in contrast, SCRs did not differ for Instruction or Block, *Fs*(1,19) = 2.55 and 0.53, *ps* = 0.13 and 0.48, η_p_^2^ = 0.21 and 0.03. Whereas no interaction of Instruction × Block was found, *F*(1,19) = 0.41, *p* = 0.53, η_p_^2^ = 0.02, exploratory analyses indicated threat-enhanced SCRs to happy faces cueing threat during the instantiation, *F*(1,19) = 6.85, *p* < 0.05, η_p_^2^ = 0.27, but not when threat was cued by angry faces in the subsequent reversal block, *F*(1,19) = 0.79, *p* = 0.38, η_p_^2^ = 0.04. No covariation effects were observed between SCRs and anxiety scores.

### Phasic heart rate changes

Overall, heart rate revealed a significant deceleration when viewing threat relative to safety cues, *F*(1,39) = 4.03, *p* = 0.05, η_p_^2^ = 0.09 (see Fig. 3C). Furthermore, there was an interaction of Time × Instruction, *F*(11,429) = 5.48, *p* < 0.01, η_p_^2^ = 0.12. Follow-up analyses were calculated separately for each time interval and indicated significant heart rate deceleration for threat relative to safety cues between 3 and 5 s after picture onset (all *ps* < 0.05). Neither the main effect Block, *F*(1,39) < 0.01, *p* = 0.98, η_p_^2^ < 0.01, nor the interactions Instruction × Block, *F*(1,39) = 0.78, *p* = 0.38, η_p_^2^ = 0.02, Time × Instruction × Block, *F*(11,429) = 1.04, *p* = 0.38, η_p_^2^ = 0.03, nor the four-way interaction by Order reached significance, *F*(11,418) = 0.65, *p* = 0.58, η_p_^2^ = 0.02.

Exploratory analyses focused separately on the different experimental orders. For the Angry-Happy order, when angry faces served as a threat cue in the instantiation block, a substantial threat deceleration was evident, *F*(1,19) = 8.98, *p* < 0.01, η_p_^2^ = 0.32, which developed over time following the threat cue onset, *F*(11,209) = 5.22, *p* < 0.01, η_p_^2^ = 0.22. However, no threat effects emerged in the subsequent reversal block when happy faces served as new threat cue, *F*(1,19) = 0.01, *p* = 0.93, η_p_^2^ < 0.01. For the Happy-Angry order, in contrast, no threat effect occurred, *F*(1,19) = 2.62, *p* = 0.12, η_p_^2^ = 0.12, nor did any interaction including Instruction reach significance, *Fs* < 1.68, *ps* > 0.20, η_p_^2^ < 0.08. No covariation effects were found between phasic heart rate changes and anxiety scores.

## Discussion

The present study examined the capability of emotional facial expressions as cues for verbally instructed threat-of-shock or safety. Also, we tested the flexibility of threat and safety associations using reversal instructions. Verbal communication about threat contingencies triggered, as expected, a pronounced pattern of psychophysiological defense reactions. This was evident in potentiated eye-blink startle reflex, enhanced skin conductance responses, and heart rate deceleration. For self-report data, interaction effects of facial expressions and verbal instructions emerged. Specifically, when smiling faces cued threat, they were rated as aversive as angry faces. In contrast, physiological responding was independent of whether the threat was cued by a happy or an angry facial expression. Moreover, reversal instructions flexibly changed defense activation, leading to relatively stable threat effects despite substantial response habituation across the experimental blocks. Interestingly, after reversal instructions, threat-potentiated startle was more pronounced in more socially anxious participants. Thus, anxious individuals did not exhibit more pronounced defense activation compared to less anxious participants, but their defense activation was more persistent.

When confronting other people’s facial expressions, which were previously learned as signals for shock threat, pronounced activation of the somatic and autonomic nervous system was observed (i.e., potentiated eye-blink startle and enhanced skin conductance responses). These findings replicate previous studies showing defense activation to visual stimuli cueing instructed threat-of-shock^24–29^. Defensive responding, however, occurred regardless of whether the threat was cued by a smiling or an angry face. Thus, the inherent valence of the threat cue (happy or angry expression) was not relevant for the acquisition of threat contingencies. This finding adds to previous research using the threat-of-shock paradigm with complex natural affective scenes^4,7,24^. For example, Bradley and colleagues^24^ observed comparable threat-potentiated startle reflex to pleasant and unpleasant pictures when these served as instructed threat cues. Moreover, when pictures were not predictive for threat-of-shock (i.e., presented within a threatening context), threat effects were found similarly pronounced for pleasant, neutral, and unpleasant pictures^4,43^. The present study extends this view to face and person perception and shows that the emotional salience of happy and angry facial expressions can be easily overridden by verbal instructions about threat contingencies. This finding contributes to the rather mixed evidence on whether human faces serve as an evolutionary prepared conditional stimulus^36,37,44,45^. Compared to pictures of snakes or spiders, the human face may be a less reliable source of threat or safety information, probably because facial expressions can be manipulated consciously and are subject to social display rules^46^.

The inherent valence of an emotional face did not interact with the verbally transmitted acquisition of threat or safety contingencies. This finding is supported by previous neuroimaging research, for instance, showing that threat instructions led to a more general sensitization of stimulus processing^32,47^, regardless of the a priori meaning of a shock cue (e.g., unpleasant or neutral social scenes). Moreover, in the present study, neither the somatic (eye-blink startle) nor the autonomic nervous system (SCR and phasic HR responses) showed an interaction between visual and instructed information. Supplementary analyses using Bayesian statistics supported these findings. Likelihood estimates of the null hypothesis (i.e., no Order × Instruction × Block interaction) indicated that the null relative to the alternative hypotheses were around 19-, 37-, and 142-times more likely for the startle reflex, SCR, and HR measures respectively. Thus, the present data lend support for the notion that the processing of visual and verbal threat information is organized in (partially) distinct neural circuitry. For example, affective modulation of the startle reflex triggered by emotional pictures is impaired in patients with right rather than left temporal lobectomy, whereas the opposite pattern can be observed when instructed threat cues are presented^48^. Interestingly, our self-report data revealed result patterns that were partly in contrast to physiological measures. Valence, arousal, and threat ratings confirmed that verbal communication about potential threats clearly induced aversive anticipations. Moreover, these threat/safety contingencies were highly malleable and reversible using subsequent instructions. In contrast to physiology, however, rating data showed that the impact of threat and safety instructions varied with the inherent valence of the facial threat/safety cue. When cueing threat, a smiling face becomes as aversive as an angry face, and both cues were highly effective in triggering defensive responding to cope with the anticipated aversive event.

Overall, reversal instructions flexibly changed threat/safety associations and the corresponding physiological response patterns. In line with previous studies, verbal instructions were highly effective at reducing defensive responding using reversing affective contingencies from threat to safety^29–31^. Similarly effective was the reversal of contingencies from safety to threat. Newly learned threat cues (previously safe), compared to the newly learned safety cues (previously threatening), were associated with lower valence and higher threat ratings. Moreover, potentiation of the startle reflex was observed for the new threat cues despite pronounced response habituation across experimental blocks. This result adds to the findings of previous research, which show that instructed threat effects may be highly persistent within and across repeated sessions, even without any aversive reinforcement^4,28^.

Interestingly, after reversal instructions, threat effects varied as a function of social and trait anxiety. Specifically, anxious participants did not exhibit more pronounced defense activation compared to less socially anxious participants but did exhibit a more persistent defense activation. From a clinical perspective, this is an important finding showing that inter-individual differences in anxiety might account for the capability to learn new safety contingencies and to reduce psychophysiological defense activation. As many fears and anxieties rely on aversive anticipations rather than experiences, the mere absence of aversive events or omission of reinforcement is not sufficient for successful fear extinction learning (e.g., in generalized anxiety disorder or social phobia)^49–51^. To optimize social communication about threats and safety in a therapeutic context, different means of social learning need to be accounted for (i.e., learning by instructions and observations)^25,52^. Building upon the present inter-individual differences in reversal learning, testing (sub-)clinical samples high in social anxiety or interpersonal disturbances might be particularly informative^53,54^. Here, the implementation of a full reversal design^29,35^ might focus on safety learning and elucidate the impact of reversed compared to maintained social safety cues.

Several noteworthy aspects of the present design and findings need to be acknowledged and should be addressed in future research. Exploratory analyses provided some indication for the hypothesis that facial emotions might differentially modulate reversal learning. Specifically, for the startle reflex during the reversal block, instruction effects were more resistant to extinction when angry rather than happy faces cued threat. This finding might result from anger-superiority in threat learning (i.e., angry faces more readily associated with threat)^9,10^ or happy-superiority^11,12^ in safety learning. For directly comparing these opposing hypotheses, the use of a non-affective threat cue condition would have been useful (i.e., neutral faces cueing threat during reversal block) and cannot be resolved with the data at hand. Future research could examine the capability of distinct non-affective social stimuli as reversed threat/safety cue. For instance, invariant facial features – such as person identity and the color of the skin – have been shown to be powerful factors that bias threat learning and can be pitted against each other (e.g., viewing other-race, but same team faces)^34,36,45^. Here, social approaches to initiate persistent reversal learning (i.e., shifting aversive contingencies to other non-social cues) may help to counteract stereotypes, social avoidance, and ostracism^35,55^. From an evolutionary perspective, it appears likely that combined variant and invariant facial information (e.g., facial expression and person identity cues)^56^ critically guide behavioral responding. For instance, an angry looking out-group member or a smiling mother might be more readily learned as a signal for threat or safety; such congruency effects in prepared learning can be tested with personalized stimulus materials (e.g., pictures of attachment figures)^57,58^. Finally, the transfer to behavioral output measures appears pertinent to test the implications and consequences of threat and safety learning in social interaction situations, for instance, regarding interpersonal trust^59^, stereotyping and social group biases^33,34^, or choice behavior in clinical settings (e.g., decision to undergo treatment)^54,60^.

In summary, verbal communication about threats might easily prime defensive response programs regardless of the inherent valence of the threat or safety cue (i.e., happy or angry facial expression). Moreover, threat effects were malleable by additional verbal instructions, and the persistence of threat effects varied with inter-individual differences in social and trait anxiety. Anxious participants did not exhibit more pronounced defense activation compared to less anxious participants but did exhibit more persistent defense activation. As threat instructions were not substantiated by the individual’s own experiences (i.e., no shocks during the experiment), these findings demonstrate the effects of mere anticipatory processes in person perception relevant to maladaptive extinction learning in anxiety disorders.

## Electronic supplementary material

Supplementary Analyses

## Data Availability

The datasets generated and analyzed during the current study are available from F.B on request.
