# Supplementary material for: Verbal instructions override the meaning of facial expressions
Source: Sci Rep. 2018 Oct 9;8:14988. doi: 10.1038/s41598-018-33269-2 (PMC6177419; doi:10.1038/s41598-018-33269-2)
Supplement: Supplementary file 1 — Supplementary Analyses [file 41598_2018_33269_MOESM1_ESM.docx]

# Supplemental material to the manuscript:

Verbal instructions override the meaning of facial expressions

by

Florian Bublatzky ^1, 2^*, Pedro Guerra ^3^, & Georg W. Alpers ^2^

^1^ Department of Psychosomatic Medicine and Psychotherapy, Central Institute of Mental Health, Medical Faculty Mannheim/Heidelberg University, Germany

^2^ Clinical Psychology and Biological Psychology and Psychotherapy, Department of Psychology, School of Social Sciences, University of Mannheim, Germany

^3^ University of Granada, Department of Personality, Spain

## Content:

Supplement 1: No modulation of the startle reflex as a function of facial expression.

Supplement 2: Bayesian Analyses testing the likelihood of null hypotheses.

Supplemental references

## Supplement 1: No modulation of the startle reflex as a function of facial expression.

Exploratory analyses tested the impact of emotional relative to neutral facial expressions on the startle reflex. To this end, averages were calculated across experimental blocks, regardless of whether facial expressions cued threat or safety. Startle responses varied as a function of Facial Expression (happy, neutral, angry), *F*(2,78) = 4.99, *p* = .01, η_p_^2^ = .11, with more pronounced amplitudes for both angry and happy compared to neutral faces, *ps* = .03 and .05, but no difference between angry and happy faces, *p* = 1.0. However, startle amplitudes for emotional faces are likely overestimated (happy and angry but not neutral faces served partly as threat cue). Thus, in a more conservative test, we compared happy and angry faces (only when cueing safety) with neutral faces (which always cued safety). For these analyses, no main effect for facial expression was observed, *F*(2,78) = 0.96, *p* = .38, η_p_^2^ = .02.

## Supplement 2: Bayesian Analyses testing the likelihood of null hypotheses.

Conventional repeated measure ANOVAS for the physiological measures (startle reflex, SCR, and HR) did not reveal any interaction effects as a function of Order × Instruction × Block, startle reflex *F*(1,38) = .73, *p* = .40, η_p_^2^ = .02, skin conductance responses, *F*(1,38) = .53, *p* = .47, η_p_^2^ = .01, heart rate changes *F*(11,418) = .65, *p* = .58, η_p_^2^ = .02 (including Factor Time: 12 levels). Based on these results the interaction hypotheses (motivational priming and incongruency hypotheses) appear unlikely and we can conclude that the emotional facial expressions (coded in the between-group factor Order of instructions: Angry-Happy vs. Happy-Angry) do no modulate threat learning.

Further information in favor of the null hypothesis (no interaction effects) is provided by Bayesian analyses (Kass & Raftery, 1995). We calculated the Bayes factors (BF) of all relevant models – Instruction, Block, Instruction+Block, Instruction+Block+Order*Order, and so on (see table below) – using JASP software. JASP is an R based software package (Wagenmakers et al., 2018; Morey et al., 2015) using Monte-Carlo sampling 10000 iterations and default prior scaling factors (for fixed effects = 0.5, random effects = 1; Rouder et al., 2012). BF inclusion scores (BF_Incl_) are reported and inform about how strongly the inclusion of one factor (e.g., Order, averaged over all models that include this factor) is supported by the data, compared to all other models (including the null-model; see table).

For the startle reflex, comparisons against the null hypotheses revealed BF_Incl_ scores suggesting meaningful effects for Block, Instruction, and the interaction Block × Instruction (i.e., BF_Incl_ > 3 indicates alternative hypothesis is at least 3 times more likely than the null hypothesis). In contrast, for the main effect of Order and the interaction Block × Instruction × Order, the according null hypotheses are at least 9.17- and 19.61-times more likely than the alternative hypotheses (i.e., likelihood of null hypotheses = 1/BF_Incl_). For the skin conductance responses, meaningful effects are suggested only for Instruction (183-times more likely than the null hypothesis). In contrast, for the interaction Block × Instruction × Order the according null hypothesis is 37.04-times more likely than the alternative hypotheses. Similarly, for the analyses of phasic heart rate changes, meaningful effects are only suggested for Instruction (BF_Incl_ = 4.906 * 10^7^), but regarding the interaction of interest Block × Instruction × Order, the null hypothesis is 142.85-times more likely. Also for rating data (threat, valence, and arousal), Bayesian analyses are in line with results observed from conventional ANOVAs.

| Model | BFInclusion: | Startle | SCR | HR | Threat | Valence | Arousal |
| --- | --- | --- | --- | --- | --- | --- | --- |
| Block |  | 4.128*10^8^ | 0.464 | 0.098 | 2.106*10^11^ | 7.311*10^13^ | 608242.676 |
| Instruction |  | 1.498*10^12^ | 183.195 | 6.777 | 1.361*10^11^ | 6.565*10^13^ | 121.482 |
| Order |  | 0.109 | 0.202 | 0.131 | 9.849*10^10^ | 5.106*10^13^ | 116.320 |
| Block × Instruction | | 4.533 | 0.481 | 0.140 | 2.486*10^11^ | 1.251*10^14^ | 5.530 |
| Block × Order | | 0.110 | 0.198 | 0.027 | 4.013*10^11^ | 1.859*10^14^ | 272.915 |
| Instruction × Order | | 0.113 | 0.200 | 0.113 | 1.908*10^11^ | 9.807*10^13^ | 7.037 |
| Block × Instruction × Order | | 0.051 | 0.027 | 0.007 | 1.396*10^12^ | 5.477*10^14^ | 6.224 |
| Note. Bayes factors (BF_incl_) of the selected models compared to all models without this factor for the different dependent measures | | | | | | | |

Taken together, regarding physiological measures, both conventional repeated measure ANOVAs (null hypothesis statistical testing) as well as Bayesian analyses confirmed that instructed threat effects did not vary as a joint function of experimental Order and Block, that is, which facial expression cued threat/safety during the instantiation and reversal blocks.

## Supplemental references:

JASP Team (2018). JASP (Version 0.9)[Computer software]. https://jasp-stats.org/

Kass, R. E., & Raftery, A. E. (1995). Bayes factors. Journal of the American Statistical Association, 90(430), 773-795.

Morey, R. D., Rouder, J. N., & Jamil, T. (2015). BayesFactor: Computation of Bayes factors for common designs. R package version 0.9, 9, 2014.

Rouder, J. N., Morey R. D., Speckman P. L., & Province, J. M. (2012). Default Bayes factors for ANOVA designs. Journal of Mathematical Psychology, 56, 356-374. DOI: 10.1016/j.jmp.2012.08.001

Wagenmakers, E. J., Marsman, M., Jamil, T., Ly, A., Verhagen, J., Love, J., ... & Matzke, D. (2018). Bayesian inference for psychology. Part I: Theoretical advantages and practical ramifications. Psychonomic Bulletin & Review, 25(1), 35-57. DOI: 10.3758/s13423-017-1343-3
